# Initial development of perpetrator confrontation using deepfake technology in victims with sexual violence-related PTSD and moral injury

**DOI:** 10.3389/fpsyt.2022.882957

**Published:** 2022-08-18

**Authors:** Agnes van Minnen, F. Jackie June ter Heide, Tilly Koolstra, Ad de Jongh, Sezer Karaoglu, Theo Gevers

**Affiliations:** ^1^Behavioural Science Institute, Radboud University Nijmegen, Nijmegen, Netherlands; ^2^Research Department PSYTREC, Bilthoven, Netherlands; ^3^ARQ Centrum‘45, Diemen, Netherlands; ^4^Academic Centre for Dentistry Amsterdam, University of Amsterdam and VU University Amsterdam, Amsterdam, Netherlands; ^5^3DUniversum, Amsterdam, Netherlands; ^6^University of Amsterdam, Amsterdam, Netherlands

**Keywords:** PTSD, moral injury, deepfake, virtual reality, therapy, prolonged exposure, EMDR therapy

## Abstract

**Background:**

Interventions aimed at easing negative moral (social) emotions and restoring social bonds – such as amend-making and forgiving—have a prominent role in the treatment of moral injury. As real-life contact between persons involved in prior morally injurious situations is not always possible or desirable, virtual reality may offer opportunities for such interventions in a safe and focused way.

**Objective:**

To explore the effects of the use of deepfake technology in the treatment of patients suffering from PTSD and moral injury as a result of being forced by persons in authority to undergo and commit sexual violence (so-called *betrayal trauma*).

**Methods:**

Two women who had experienced sexual violence underwent one session of confrontation with the perpetrator using deepfake technology. The women could talk *via* ZOOM with the perpetrator, whose picture was converted in moving images using deepfake technology. A therapist answered the questions of the women in the role of the perpetrator. Outcome measures were positive and negative emotions, dominance in relation to perpetrator, self-blame, self-forgiveness, and PTSD-symptom severity.

**Results:**

Both participants were positive about the intervention. Although they knew it was fake, the deepfaked perpetrator seemed very real to them. They both reported more positive and less negative emotions, dominance in relation to the perpetrator and self-forgiveness, and less self-blame and PTSD-symptoms after the intervention.

**Conclusion:**

Victim-perpetrator confrontation using deepfake technology is a promising intervention to influence moral injury-related symptoms in victims of sexual violence. Deepfake technology may also show promise in simulating other interactions between persons involved in morally injurious events.

## Introduction

Moral injury is as a psychosocial condition that may develop after committing, failing to prevent, or witnessing acts that transgress deeply held moral beliefs and expectations and that take place in high stakes situations in which a person is harmed by another (1, 2). Such transgressions, also known as potentially morally injurious experiences (PMIE's), can be divided into perpetration-based and betrayal-based experiences, with betrayal referring to being subjected to another's transgressive behavior, especially that of a person in power, a leader or a trusted authority (3). PMIE exposure may lead to the development of a range of symptoms including negative attributions, negative moral emotions such as guilt and shame, social withdrawal, failure to forgive oneself and others, self-handicapping behaviors and PTSD (1). Moral injury is perceived as being distinct from, but associated with PTSD. Overlap between moral injury and PTSD may be stronger where a PMIE is both morally injurious as well as meets the A-criterion for PTSD (3).

Given that PMIE's involve a morally transgressive interaction between different people - in the roles of perpetrator, victim, helper, bystander and authority - moral injury can be perceived as a form of interpersonal trauma e.g., (4). The suffering caused by moral injury is interpersonal, centering around negative moral emotions and social withdrawal (1). Consequently, the interventions administered to alleviate or heal moral injury are often interpersonally-focused. Making amends, seeking or offering forgiveness, and acting on important social values are among the interventions recommended for treating moral injury e.g., (5). It is believed that through these interventions, interpersonal connections can be restored, negative moral emotions alleviated, and negative attributions considering self or others, corrected.

Interpersonally-focused interventions to alleviate moral injury can be conducted face-to-face or imaginarily. Previous studies found that face to face victim-perpetrator confrontations generally lead to positive outcomes for both victims and perpetrators (6). However, face-to-face contact between persons involved in PMIE's may not always be possible nor desirable. People involved in PMIE's may have died, access to remaining family members may be prohibited, the PMIE's may have taken place in far-away places that are no longer accessible, or disclosing PMIE's may be restricted. Furthermore, it is conceivable that specifically with regard to betrayal trauma, the victim is too fearful to confront the perpetrator, the perpetrator is unemphatic, or contact with the perpetrator is considered unsafe. In such cases, imaginary conversations, such as an imaginal dialogue with a benevolent moral authority, may be used (1). During such a dialogue, the patient may share their morally injurious experiences and consequent suffering with the moral authority, followed by an imaginary, supportive response by the moral authority.

Participation in an imaginary dialogue requires imaginary skills which some patients may not master. To solve this issue, in recent years virtual reality environments are being developed that may simulate interpersonal interactions and thus promote interpersonal healing or closure. Recently, virtual reality applications have been suggested to be a good and safe alternative for a live interaction in a therapeutical setting, for example in relation to prolonged grief (7).

Recently, AI models (deepfakes) have been developed to generate and manipulate fake faces that look almost identical to real people. Due to the photorealistic content, deepfake technology (e.g., face or lip synching) can be a suitable alternative for live interventions. Deepfake therapy (https://deepfake-therapy.com/) is an online communication platform to enter into conversation with people through self-controlled video animations using deepfake technology *via* Zoom.

This paper describes the use of newly developed deepfake therapy technology with two patients who had been morally injured through sexual violence. Sexual violence has been conceptualized as a form of betrayal trauma, both within a military context e.g., (8, 9) and a civilian context (10). Like other forms of betrayal trauma, it often involves a betrayal of trust by persons of power or authority and may lead to strong negative moral emotions and cognitions of shame, guilt and anger. In the cases discussed in this paper, patients were abused by a boss and a group of older boys, respectively; one patient was also forced into perpetration with other children. Both patients were treated using the innovative deepfake therapy platform, after evidence-based trauma-focused therapy had been of limited effect on their negative moral emotions and cognitions. Because moral injury involves different domains including negative attributions (such as self-blame), negative moral emotions (such as guilt), social withdrawal, inability to forgive, and PTSD symptoms, we measured self-blame and self-forgiveness, PTSD symptoms, empowerment and negative and positive emotions before and after the intervention. Given that currently, most instruments of moral injury focus on the experiences of military veterans, no integral moral injury questionnaire was used.

## Methods

### Procedure

Both patients had repeatedly been exposed to childhood sexual violence, and were diagnosed with PTSD as measured with the Clinician-Administered PTSD Scale for DSM-5 (CAPS-5; Dutch version (11). They received a brief intensive trauma-focused treatment program lasting 8 days. This treatment program contains two first line trauma-focused treatments for PTSD (eight sessions prolonged exposure and eight sessions EMDR therapy), in combination with physical activity and psycho-education. For more detailed information about this treatment program, we refer to (12). After this trauma-focused treatment program, the patients did not fulfill the diagnostic criteria for PTSD (CAPS-5) anymore. However, it appeared that at 6-month follow-up they still struggled with negative moral emotions and cognitions about themselves in relation to the perpetrator (such as anger and self-blame), and therefore, we invited them to undergo a novel intervention using artificial intelligence (“deepfake”) technology. They signed an informed consent form, and both 1 week before the intervention and 1 week after the intervention they filled in the outcome measures at home; that is, the Posttraumatic Cognitions Inventory (PTCI), the Heartland Forgiveness Scale, and the PTSD Checklist (PCL-5). In addition, directly before and after the deepfake intervention, the patients filled in two state measures: the Positive and Negative Affect Schedule (PANAS) and the Social Comparison Scale. The deepfake intervention was situated in a lab at 3DUniversum (spin-off of the University of Amsterdam).

## Instruments

### Outcome measures (one week before and after the intervention)

#### Self-blame

The Posttraumatic Cognitions Inventory PTCI; (13, 14) is a self-report measure with 33 items assessing trauma-related negative cognitions. We only report the data of the subscale self-blame (five items). No cutoff scores are available, but participants with trauma and no PTSD have a median score of 1.00, and with PTSD 3.20. The PTCI has good internal consistency and validity.

#### Self-forgiveness

The Heartland Forgiveness Scale (15) is a self-report measure with 18 items assessing forgiveness. We report the self-forgiveness scale data which contains six items. The range of scores is 6–42, and scores above 29 are considered an indication that one is usually forgiving of oneself. The scale has good reliability and validity.

#### PTSD symptoms

The PTSD Checklist PCL-5; (16) was used as a self-report measure to measure the severity of the PTSD symptoms. It consists of 20 items (range total score 0–80). Generally, a cutoff score of >33 is used as an indication of PTSD. The PCL-5 has high internal consistency and good validity (17).

### State measures (immediately before and after the intervention)

#### Empowerment

We used (an adapted version of) the Social Comparison Scale (18) to measure how the patient relates to the perpetrator in terms of power and strength. This measure contains 11 items (range 1–10). Examples of items are: “*In relationship to the perpetrator I feel…”* with bipolar response categories between “weak” (1) and “strong” (10), and between “without self-confidence” (1) and “full of self-confidence” (10). The scale has been found to be reliable. No cutoff-scores are available, but a clinical group scored 38.90, while a control group scored 64.67 on the total scale (ranging from 11 to 110).

#### Positive and negative emotions

The Positive and Negative Affect Schedule (PANAS) is a self-report questionnaire containing 10 items about positive and 10 items about negative emotions at a specific moment (19). The range for both scales is 10–50. No cutoff scores are available, but in the original study mean scores for the Positive Affect Score was 33.3 and for the Negative Affect Score 17.4.

#### Intervention

**Figure 1:**
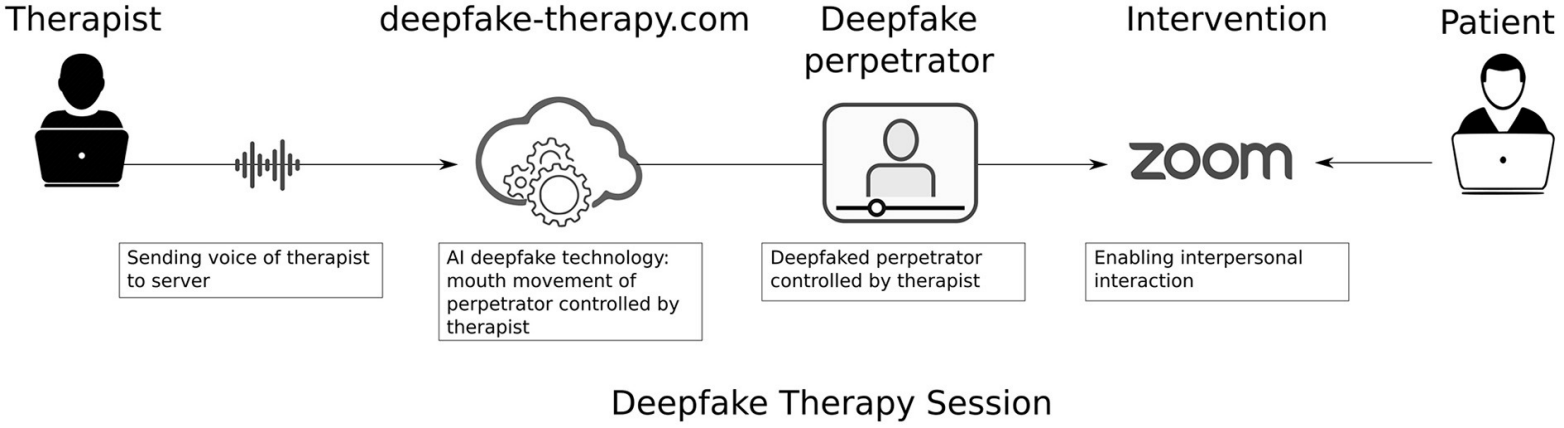
Overview of deepfake therapy session.

The women received one session of 90 min, and could talk *via* ZOOM with the deepfaked perpetrator (see Figure 1). Before the intervention the women sent a picture of the perpetrator to the deepfake therapy platform that converted this picture in a video of the perpetrator using deepfake technology. The patient was in one room sitting behind a laptop, and was connected *via* ZOOM with the deepfaked perpetrator who was sitting behind a laptop in another room. The role of the perpetrator was fulfilled by a clinical psychologist who was trained in working with traumatized patients. The therapist answered the questions of the women in the role of the perpetrator. During the Zoom session, the deepfaked face of the perpetrator was controlled by the voice of the therapist. The voice of the therapist causes the deepfaked face of the perpetrator to make mouth movements that mimic the therapists' voice. In this way, the therapist controls the deepfaked face directly and live, enabling an interactive conversation. The voice of the therapist was not deepfaked, i.e., the voice of the deepfaked perpetrator was the voice of the therapist.

Some weeks before the intervention, the patients could prepare themselves at home, and they were instructed that they could say and do anything they wanted, and could interrupt or stop the intervention at any time they wished. The preparation was facilitated with a standard list of questions and themes that was specifically developed for use with the deepfake technology, and was loosely based on questions that are used within the setting of real-life victim-perpetrator confrontations.

Examples of the questions that were included are; What do you want to say to the perpetrator about your feelings, or about the (emotional) consequences of the traumatic event(s) on your daily functioning? What do you want to ask the perpetrator about choosing you as a victim? During the intervention another clinical psychologist was present whom the patients could consult at any time.

The therapist in the role of the perpetrator was instructed to act as an empathic person, and to reduce self-blame and enhance self-forgiveness of the victim. The therapist did not know what the victim wanted to say or ask, and his reactions were facilitated with a manual containing standard messages. Examples of the theme of these messages were; I did not realize what I did and how much impact this would have for you and I am to blame, you are not to blame.

## Results

### Case 1 Jill

The first patient was a 36-years-old woman, who was sexually and physically abused during her teenage years. When she was 15 years old, she took an after-school job in a local shop. She confided in her boss about her emotions regarding a friend who was terminally ill. Her boss, who was older, sexually assaulted her repeatedly when she was at work, humiliated her, and was physically abusive. She felt betrayed that he assaulted her during that vulnerable time in her life while she trusted him. At that time, she never told anyone about the abuse, and she pretended that everything was okay. She was diagnosed with PTSD, avoided the shop the perpetrator worked in, was hypervigilant, and had negative self-related cognitions and emotions. Prior to this intensive treatment program, she received EMDR therapy twice, without any result. She did not use any medication. During the intensive trauma-focused treatment, including prolonged exposure and EMDR therapy, she was confronted with the memories of the sexual violence and a picture of the perpetrator. Also, negative moral cognitions and emotions, such as guilt, shame and self-blame were successfully targeted during these sessions, for instance with cognitive processing and cognitive interweaves. Her PTSD-symptoms decreased and at posttreatment the PTSD diagnosis was in remission. However, she still could not forgive herself for not telling anyone about the sexual violence, and blamed herself for not having done the “right” things at that time, for instance tell anyone or leave the job. She also felt anger, because she felt betrayed by the perpetrator. Her motivation to confront herself with the perpetrator using deepfake, was that the perpetrator was still working in her neighborhood, and she still avoided the shop where she was abused, being fearful to be confronted with him. It felt like he still had power over her.

During the deepfake confrontation, Jill was emotional and cried. She was nervous and fearful to be confronted with the perpetrator. In the beginning of the confrontation, she was trembling and seemed confused, had trouble finding the right words, and avoided eye-contact with the perpetrator. As the conversation progressed, she expressed more anger and was able to clearly state her opinion of him.

Jill: “Uhm…When I was driving to this appointment…I was thinking…. This is difficult for me…I wanted to say some things to you…uhm…how you…uhm…I don't know, how this could have happened, I don't know, uhm…. I think my question is: why did you do this? You knew that my friend was going to die and that I was vulnerable, and despite that, you assaulted me”.

Perpetrator: “Yes, looking back I realize that I took advantage of that situation, and I was selfish by doing that. It had nothing to do with you, it was not your fault”.

Jill: “Indeed, I have learned in therapy that it is your fault, and only yours ……You don't have a clue about how this affected my life”.

Perpetrator: “You are right about that, I never realized what I did to you, I was only focused on myself. I am alarmed to hear how much impact this had on you and your life, this should never had happened”.

Jill: “I will never forgive you for what you did to me. I don't want revenge or something, but I really hope you stay away from me (is crying). That would give me peace. When my burden is symbolized in a brick, I would like to give this brick to you, so that you feel this burden every day from now on, and that I can get rid of it (emotional)”.

Perpetrator: “Yes, if someone has to suffer from this, it should be me, you're right”.

Jill: “I want to feel strong when I am confronted with you, I want to feel bigger than you, I see you as the loser in this situation. You are weak”.

Perpetrator: “I feel weak, indeed, and you are strong and brave. I admire you, how you coped with it and rebuilt your life”.

Jill: “I want to let it go. I am happy now”.

Perpetrator: “You deserve that”.

### Patient perspective

This deepfake experience was emotional for me, and it helped me a lot to be confronted with him, and to experience that he was no longer a man to be afraid of. It felt different than imaginal exposure, looking at his picture, or writing a letter to him, as I did in my previous therapies. His image was “alive” now, and he felt real to me. It was scary to speak with him in the beginning, but when I got used to it, I felt in control. I am much stronger now, and I pity him. I realized that he's the loser, not me. And even though I already knew that I did not have to blame myself, I now really felt it deep inside of me. After this session I visited the shop where he worked and where it all happened, and I no longer was afraid to do so.

### Outcome and state measures Jill

See Table 1 for an overview. Immediately after the deepfake session, Jill showed more positive and less negative emotions, and an increase in self-empowerment. 1 week after the deepfake session she showed less self-blame, more self-forgiveness, and a further decrease in PTSD symptoms.

**Table 1:** Outcome and state measures during and after the deepfake session.

		**Pre**	**Post**	**(indications of) normal range of score**
**Jill**	
	*Outcome measures*	
	Self-blame	4.50	2.00	<1.00
	Self-forgiveness	23	29	>29
	PTSD symptoms	11	4	<33
	*State measures*	
	Empowerment	59	95	>65
	Positive emotions	27	41	>33
	Negative emotions	21	8	<17
**Meg**	
	*Outcome measures*	
	Self-blame	3.20	1.20	<1.00
	Self-forgiveness	23	32	>29
	PTSD symptoms	47	16	<33
	*State measures*	
	Empowerment	52	94	>65
	Positive emotions	33	40	>33
	Negative emotions	27	18	<17

### Case 2 Meg

Meg was a 48-years-old woman. As a child and teenager, she was repeatedly sexually abused by a group of older boys who also forced her to sexually abuse other children. Consequently, Meg felt that this group made her a perpetrator as well as a victim. She was diagnosed with PTSD, avoided to think and talk about the sexual abuse, had severe negative cognitions about herself, showed angry outbursts and had sleep problems. Prior to this intensive treatment program, she received several EMDR therapy sessions and she is using sertraline 100 mg/day, with no effect. During the prolonged exposure and EMDR therapy sessions of the intensive treatment program, she was repeatedly confronted with her memories of the abuse and pictures of the abusers. Negative moral emotions such as shame and guilt were successfully targeted, for instance by imaginably expressing anger to the perpetrator. After the trauma-focused treatment program, her PTSD symptoms were in remission. However, at 6 months follow-up she relapsed, and was again diagnosed with PTSD. She explained her relapse by the fact that she could not get over the feeling that she failed to stand up for herself and felt guilty about abusing other children.

During the deepfake intervention, she wanted to confront the main perpetrator, the leader of the group. Her main question was why he had chosen her, and whether he realized what he had done. Meg was really angry at the perpetrator, and the more he answered her questions and explained the situation, the angrier she got.

Meg: “What in the world were you thinking as a 15-year-old boy when you assaulted me, a 4-year-old little innocent girl?”.

Perpetrator: “I did not realize at that time what I did to you. I was only involved with myself. It alarms me to hear how much impact it had on your life”.

Meg: “Do you realize what you did to a 4-year-old child?”.

Perpetrator: “At that time? No. I now realize that it was disgusting what I did”.

Meg: “Did you carefully plan this and choose me?”.

Perpetrator: “No, it could have been any child”.

Meg: “I was only 8 years old when you forced me to have sex with another child. You were an adult at that time. Why did you do this? I still feel guilty about it”.

Perpetrator: “Yes, you are right, I should never have done this, I am sorry. And I forced you to do this, it was not your fault”.

Meg: “Do you know what you caused? I struggled with this my whole life, for 40 years now”.

Perpetrator: “I am sorry to hear that. I want you to know that it was not your fault, it was my fault, I am the guilty one. It was disgusting what I did. I feel very bad about it, every day”.

Meg: “Well, you should feel bad. But it pisses me off that you feel self-pity now. I suffered more than you did, I always feel scared, I feel dirty every day”.

Perpetrator: “I understand that. I believe that you are really strong that you've survived this. I feel like a loser, but I've learned from it”.

Meg: “You are an asshole, you destroyed so many lives, you ruined my life. You caused so much damage. I hope you have a miserable life, and I hope that I never have to see you again. I don't want to feel bad about myself anymore because of what *you* and *only you* did. I hope you'll drop dead” (Meg closes the laptop with a smash).

### Patient perspective

**“**This deepfake experience really had an impact on me, because for the first time I was able to stand up for myself, and express my anger toward him. Although I already expressed my anger in imagination during previous trauma-focused treatment sessions, this deepfake setting made it more real to me, and therefore, it's a very powerful tool. Although I knew it was fake, I really had the feeling that I was talking to him. I felt scared, and afterwards I felt my sweaty back, but nevertheless, for the first time I felt the power to overrule him. This intervention made me realize that it had nothing to do with me, and I was just a random victim, it could have been anyone else. I did nothing wrong. They did. If I could choose, I would have had more deepfake sessions with all the perpetrators to tell them how wrong they were, and that they could not hurt me anymore”.

### Outcome and state measures Meg

See Table 1 for an overview. Immediately after the deepfake session, Meg showed more positive and less negative emotions, and an increase in empowerment. 1 week after the deepfake session she showed less self-blame, more self-forgiveness, and her PTSD-symptoms were in remission.

## Discussion

In this article two cases were presented using deepfake technology in the treatment of sexual violence-related moral injury and PTSD. The deepfake intervention aimed at overcoming negative moral emotions and cognitions, and resulted in less self-blame and more self-forgiveness. Also, PTSD symptoms decreased, especially negative cognitions and avoidance behavior. What is more, self-empowerment increased, which is important, given that due to a perceived power imbalance, many victims of sexual violence have a lack of empowerment when confronted with (reminders of) the perpetrator.

Both patients were satisfied with this intervention, were able to tolerate this 90-min session, and would highly recommend it to others. Although they were aware that they did not actually talk with the perpetrator, they both experienced the deepfake intervention as a real-life confrontation with the perpetrator, with real-time interaction.

Therefore, they experienced it as a double valuable add-on intervention to techniques that are often used in other therapies, such as confrontation with static stimuli like photos of the perpetrator, (during exposure therapy), or imaginal confrontations (during EMDR-therapy sessions). One advantage of deepfake confrontation between a victim and a perpetrator as opposed to real life confrontation, is that with deepfake, the perpetrator (the therapist) is always responding with empathy toward the victim, and therefore negative reactions, such as revictimization, can be avoided and safety of the victim is guaranteed. It is however important to also guide the patient after this intervention, especially when the patient is planning to have a real-life confrontation with the perpetrator, to prevent possible adverse effects.

Trauma-focused treatments such as prolonged exposure have been shown to be effective in reducing PTSD symptoms in patients with moral injury [e.g., (20, 21)]. However, in some cases, like the cases presented here, additional interventions may be needed to specifically target negative moral emotions and cognitions. Other treatments that specifically focus on relieving moral injury are promising [e.g., Adaptive Disclosure; (22)]. However, in the above-mentioned studies, the outcome measure was limited to PTSD-symptoms including trauma-related guilt, while these cases are one of the first that specifically address changes in negative moral cognitions and emotions such as self-forgiveness, empowerment, and self-blame.

Other strengths are that the deepfake intervention was brief (one session), is a safe intervention, and had strong effects. The intervention may be adapted to the different positions that patients may have had during the PMIE as someone who committed a transgressive act, witnessed or failed to prevent such an act, or fell victim to such an act. Therefore, it is a promising new treatment technique for moral injury. However, it may not be suitable for every patient, and case by case careful considerations have to be made. Also, long term effects are unknown, and we do not know whether our results are generalizable to participants suffering from other trauma types. In addition, ethical issues have to be considered, for instance sharing private information with private companies. In the technology we used, all materials including photos could be included or deleted by the therapist.

More studies are needed, especially controlled studies [see also (23)] that include moral injury questionnaires. We conclude that confrontation with perpetrators using deepfake technology is a promising (add-on) treatment tool for patients with moral injury.

## Data availability statement

The raw data supporting the conclusions of this article will be made available by the authors, without undue reservation.

## Ethics statement

Ethical review and approval was not required for the study on human participants in accordance with the local legislation and institutional requirements. The patients/participants provided their written informed consent to participate in this study. Written informed consent was obtained from the individual(s) for the publication of any potentially identifiable images or data included in this article.

## Author contributions

AM and TK designed the study and wrote the protocol. AM and FH wrote the first draft of the manuscript. All authors discussed the results, critically revised this manuscript, and approved the submitted version of this manuscript. All authors contributed to the article and approved the submitted version.

## Conflict of interest

TG and SK are co-founders of 3DUniversum, Amsterdam, Netherlands. SK is also employed at 3DUniversum. The remaining authors declare that the research was conducted in the absence of any commercial or financial relationships that could be construed as a potential conflict of interest.

## Publisher's note

All claims expressed in this article are solely those of the authors and do not necessarily represent those of their affiliated organizations, or those of the publisher, the editors and the reviewers. Any product that may be evaluated in this article, or claim that may be made by its manufacturer, is not guaranteed or endorsed by the publisher.
