# Corrigendum: Initial development of perpetrator confrontation using deepfake technology in victims with sexual violence-related PTSD and moral injury

**DOI:** 10.3389/fpsyt.2023.1157425

**Published:** 2023-02-14

**Authors:** Agnes van Minnen, F. Jackie June ter Heide, Tilly Koolstra, Ad de Jongh, Sezer Karaoglu, Theo Gevers

**Affiliations:** ^1^Behavioural Science Institute, Radboud University Nijmegen, Nijmegen, Netherlands; ^2^Research Department PSYTREC, Bilthoven, Netherlands; ^3^ARQ Centrum‘45, Diemen, Netherlands; ^4^Academic Centre for Dentistry Amsterdam, University of Amsterdam and VU University Amsterdam, Amsterdam, Netherlands; ^5^3DUniversum, Amsterdam, Netherlands; ^6^University of Amsterdam, Amsterdam, Netherlands

**Keywords:** PTSD, moral injury, deepfake, virtual reality, therapy, prolonged exposure, EMDR therapy

In the published article, there was an error in the **Conflict of interest**: “The authors declare that the research was conducted in the absence of any commercial or financial relationships that could be construed as a potential conflict of interest.” The correct conflict of interest appears below:

