# Supplementary material for: Initial development of perpetrator confrontation using deepfake technology in victims with sexual violence-related PTSD and moral injury
Source: Front Psychiatry. 2022 Aug 18;13:882957. doi: 10.3389/fpsyt.2022.882957 (PMC9435301; doi:10.3389/fpsyt.2022.882957)
Supplement: Supplementary file 1 [file Table_1.docx]

*Table 1. Outcome and state measures during and after the deepfake session*

|  |  | Pre | Post | (indications of) normal range of score |
| --- | --- | --- | --- | --- |
| Jill |  |  |  |  |
|  | *Outcome measures* |  |  |  |
|  | Self-blame | 4.50 | 2.00 | <1.00 |
|  | Self-forgiveness | 23 | 29 | >29 |
|  | PTSD symptoms | 11 | 4 | <33 |
|  | *State measures* |  |  |  |
|  | Empowerment | 59 | 95 | >65 |
|  | Positive emotions | 27 | 41 | >33 |
|  | Negative emotions | 21 | 8 | <17 |
| Meg |  |  |  |  |
|  | *Outcome measures* |  |  |  |
|  | Self-blame | 3.20 | 1.20 | <1.00 |
|  | Self-forgiveness | 23 | 32 | >29 |
|  | PTSD symptoms | 47 | 16 | <33 |
|  | *State measures* |  |  |  |
|  | Empowerment | 52 | 94 | >65 |
|  | Positive emotions | 33 | 40 | >33 |
|  | Negative emotions | 27 | 18 | <17 |
